# Interaction of *Talaromyces marneffei* with free living soil amoeba as a model of fungal pathogenesis

**DOI:** 10.3389/fcimb.2022.1023067

**Published:** 2022-10-03

**Authors:** Kritsada Pruksaphon, Joshua D. Nosanchuk, Patcharin Thammasit, Monsicha Pongpom, Sirida Youngchim

**Affiliations:** ^1^ Department of Microbiology, Faculty of Medicine, Chiang Mai University, Chiang Mai, Thailand; ^2^ Departments of Microbiology and Immunology and Medicine, Albert Einstein College of Medicine, Bronx, NY, United States

**Keywords:** *Talaromyces marneffei*, dimorphic fungus, *Acanthamoeba castellanii*, phagocytosis, melanin

## Abstract

*Talaromyces* (*Penicillium*) *marneffei* is an important dimorphic mycosis endemic in Southeast Asia and Southern China, but the origin and maintenance of virulence traits in this organism remains obscure. Several pathogenic fungi, including *Cryptococcus neoformans*, *Aspergillus fumigatus, Blastomyces dermatitidis*, *Sporothrix schenckii*, *Histoplasma capsulatum* and *Paracoccidioides* spp. interact with free living soil amoebae and data suggests that fungal pathogenic strategies may emerge from environmental interactions of these fungi with ubiquitous phagocytic microorganisms. In this study, we examined the interactions of *T. marneffei* with the soil amoeba *Acanthamoeba castellanii*. *T. marneffei* was rapidly ingested by *A. castellanii* and phagocytosis of fungal cells resulted in amoeba death after 24 h of contact. Co-culture also resulted in a rapid transition for conidia to the fission-yeast form. In addition, well-established virulence factors such as melanin and a yeast specific mannoprotein of *T. marneffei* were expressed during interaction with *A. castellanii* at 37°C. Our findings support the assumption that soil amoebae environmental predators play a role in the selection and maintenance of particular features in *T. marneffei* that impart virulence to this clinically important dimorphic fungus in mammalian hosts.

## Introduction

Most of the human pathogenic fungi found in the environment live in ecological niches defined by soils, trees, and decaying vegetation. Soils are harsh environments, and soil-dwelling microbes must constantly adapt to changing conditions (Torsvik and Øvreås, 2008). Soil microbes occupy environments where there is brutal competition for nutrients. In addition to these nutritional and physical stresses, soil-dwelling microbes must cope with predators in the form of amoebae and other protists that try to feed on bacteria and fungi (Jousset, 2012; Siddiqui and Khan, 2012). Consequently, soil-dwelling microbes must develop ways to escape phagocytosis and/or survive ingestion through mechanisms that support intracellular survival.

*Talaromyces* (formerly *Penicillium*) *marneffei* is a thermally dimorphic fungus found in the tropical and subtropical regions of Asia and is the causative agent of talaromycosis (Vanittanakom et al., 2006; Limper et al., 2017). Talaromycosis primarily affects people with advanced HIV disease and other immunocompromised conditions, but it is becoming increasingly reported in immunocompetent individuals. Infection with *T. marneffei* is presumably acquired *via* the respiratory route and clinically presents as a disseminated illness with fever, anemia, weight loss, lymphadenopathy, hepatosplenomegaly, skin lesions and pancytopenia (Vanittanakom et al., 2006; Cooper and Vanittanakom, 2008).

The majority of pathogenic fungi are saprophytic because they are free-living and do not require an animal host to reproduce. Similarly, several dimorphic fungi are significant human pathogens, but the origin and maintenance of virulence in these organisms is unknown, as contact with a mammalian host is not needed for fungal survival. Amoebae are an extremely diverse group of eukaryotic microorganisms that constitute a major class of phagocytic organisms in soils. *Acanthamoeba* are free-living, ubiquitous ameba that occurs in trophozoite and cyst stages during their life cycle. They can feed on diverse bacteria and fungi, which is known as “mycophagy” (Jousset, 2012; Siddiqui and Khan, 2012). *Acanthamoeba castellanii*, one of the mycophagous amoebae, has emerged as a prominent model system for the study of the evolution of virulence in many types of microorganisms, including fungi, leading to the hypothesis that aspects of the virulence of certain human pathogenic fungi arose by chance as a result of amoeboid predator selection in soils (Casadevall et al., 2003). For instance, the interactions of *Cryptococcus neoformans* and macrophages, slime molds (Steenbergen et al., 2003), and amoebae (Steenbergen et al., 2001) are remarkably similar, indicating that fungal pathogenic strategies may emerge from environmental interactions with phagocytic microorganisms. After this seminal work with *C. neoformans*, subsequent studies found that the interactions of *A. castellanii* and dimorphic fungi such as *Histoplasma capsulatum*, *Blastomyces dermatitidis*, *Sporothrix* spp., and *Paracoccidioides* spp., similarly reproduced interactions seen with macrophages (Steenbergen et al., 2004; Singulani et al., 2018; Albuquerque et al., 2019; Lemos Tavares et al., 2020). *A. castellanii* not only serves as a host system for those dimorphic fungi, but also for *Aspergillus fumigatus* (Van Waeyenberghe et al., 2013), *Cryptococcus gattii* (Malliaris et al., 2004), and entomogenous fungi (Bidochka et al., 2010).

Given that *T. marneffei* is a dimorphic fungus present in the soil environment (Vanittanakom et al., 2006), we hypothesized that *A. castellanii*, a ubiquitous environmental phagocytic predator, may exert selection on *T. marneffei*. Therefore, we explored the interaction of *T. marneffei* with *A. castellanii* and found that *T*. *marneffei* effectively expressed virulence characteristics, which suggests that the pathogen has exploited environmental amoebae as a training ground for mammalian infection.

## Materials and methods

### Microorganisms and culture conditions

*Talaromyces marneffei* ATCC 200051 was obtained from a bone marrow sample of an AIDS patient at the Central Laboratory, Maharaj Nakorn Chiang Mai Hospital, Thailand (Hamilton et al., 1999). The primary isolate was kept in 20% glycerol at -80°C. The fungus was grown in the mycelial phase on Potato dextrose agar (PDA; Difco) for 7 days at 28°C. Conidia were isolated by adding 5 ml of sterile phosphate buffer saline (PBS, 0.02 M, pH 7.2) onto the surface followed by gentle scraping of the mycelia growth with a cotton swab. The conidia were collected by filtration through sterile glass wool, centrifuged at 5,000 g for 15 min, and then washed three times with sterile PBS. In addition, we cultivated a clinical isolate of *Candida albicans*, identified using molecular techniques, which was received from the fungal culture collection of the laboratory of pathogenic fungi, at Maharaj Nakorn Chiang Mai Hospital, Chiang Mai, Thailand. *C. albicans* was grown on Sabouraud dextrose agar (SDA) for 24 h at 28°C, and then harvested by centrifugation at 5,000 g for 15 min and washes three times with PBS.

*A. castellanii* strain 30324 (trophozoite stage) was acquired from the ATCC. This strain was cultured at 28°C in peptone-yeast extract-glucose broth (PYG; ATCC medium 354) in the dark as described (Steenbergen et al., 2004). For experimental use and routine maintenance, *A. castellanii* was cultured as adherent cells in PYG medium at 28°C for 5-7 days in 75-cm^2^ culture flasks (Bozue and Johnson, 1996). *A. castellanii* was harvested by tapping the flasks, centrifuged at 2,500 rpm for 10 min, and suspended in sterile PBS. Cell counts were obtained using a modified Fuchs-Rosenthal chamber, and viability was evaluated by trypan blue staining, and the initial viability was always greater than 98%.

### Labeling *T. marneffei* conidia with fluorescein isothiocyanate

*T. marneffei* conidia were labeled with 0.1 mg/ml fluorescein isothiocyanate (FITC, Sigma-Aldrich, St. Louis, USA) in 0.1 M carbonate buffer (pH 9.0) at 4°C overnight with shaking. Labeled conidia were then washed three times with PBS containing 0.1% (v/v) Tween 20, and then counted under fluorescence microscope (Pruksaphon et al., 2020). The labeling method had no effect on viability as determined by colony-forming units (CFU) counts on PDA.

### Phagocytosis assay

*A. castellanii* cells were removed from tissue culture flasks, washed with PBS, and counted with a hemocytometer. The cells were suspended to 10^6^ cells/ml in PBS, added to 6-well plates at 10^6^ cells/well, and allowed to adhere for 2 h at 28°C before the addition of fungal cells*, T. marneffei* and *C. albicans* at a 1:10 effector-to-target ratio. The plates were spun at 500 rpm for 10 min to synchronize conidia exposure to the amoebal cells and then the samples were incubated at 37°C. After allowing phagocytosis to occur for 2 h, the media containing non-adherent, non-phagocytosed conidia was removed, and the cells were rinsed three times with PBS. The samples were fixed with ice-cold methanol for 30 min at 4°C and then the wells were scraped to harvest the amoebae. To ascertain a percentage of phagocytosis, the number of *A. castellanii* with internalized conidia per total number of *A. castellanii* cells (with and without *T. marneffei* conidia) was determined. Internalization of conidia detected with bright-field microscopy was confirmed with fluorescence visualization of internalized *T. marneffei* using a Nikon Eclipse 50i microscope (Nikon, Tokyo, Japan). The percentage of phagocytosis was determined by counting the number of *A. castellanii* with internalized conidia per 100 amoeboid cells and displaying the results as a percentage of phagocytosis. All tests were performed in triplicate. Co-cultures of *C. albicans* with *A. castellanii* were similarly performed and the results compared to the study of *T. marneffei.*

### Amoeba killing assay

Trypan blue exclusion assays were applied to determine the number of viable *A. castellanii* cells at different times, 0, 24, 48 and 72 h. Amoebae and fungal cells, *T. marneffei* or *C. albicans*, were incubated in PBS in 24-well plates at a 1:10 ratio. At each time interval, the medium was aspirated and the cultures were incubated with a 1:10 dilution of trypan blue in PBS. The 24-well plates were viewed at a magnification of ×100, and the percentage of dead amoebae was determined by counting the number of amoeba cells unable to exclude the dye per total amoebae counted. At each time interval, five wells per culture condition were counted and experiments were repeated at least one additional time on different days.

### Co-culture experiments with *T. marneffei* and *A. castellanii*

*A. castellanii* were suspended to 10^7^ cells/ml in PBS, and 1 ml was added to 12-well plate. *T. marneffei* conidia were then added to the acclimated cultures of *A. castellanii* at a 1:10 effector-to-target ratio and incubated at 37°C. At 0, 24, 48 and 72 h, the number of viable yeast cells was determined by count colony-forming units (CFU). As a control, the conidia were incubated in PBS. At each time interval, the plates were placed on ice for 10 min to loosen the cells from the bottoms of the plates. *A. castellanii* cells were lysed by shear stress induced by pulling the suspension through a 27-gauge needle (Nipro, Osaka, Japan) five to several times (Moffat and Tompkins, 1992). Fungal viability was unaffected by this procedure, as determined by comparison of initial hemocytometer determinations and CFU counts. For each well, serial dilutions were plated on PDA and incubated at 28°C for 48 h. At each time, a minimum of four tissue culture wells per isolate were used to determine CFU, and each experiment was repeated at least one time. Furthermore, co-cultures of *C. albicans* with *A. castellanii* were similarly performed and the results compared to the study of *T. marneffei*.

### Transmission electron microscopy

TEM was used to examine the intracellular compartment of *T. marneffei* within *A. castellanii*. Plastic adherent *A. castellanii* monolayers containing 2×10^6^/well in 24-well tissue culture plate were infected with *T. marneffei* at a multiplicity of infection (MOI) of 10. After 2 h of incubation at 37°C, amoeba infected with *T. marneffei* were removed by using rubber policeman and fixed with 2.5% glutaraldehyde in 0.1 M cacodylate at room temperature overnight. The samples were prepared for electron microscopy as described (Steenbergen et al., 2001). The samples were mounted with uranyl acetate and viewed in a JEOL JEM-2010 transmission electron microscope.

### Determination of *T. marneffei* phase transition in *A. castellanii* using an indirect immunofluorescence assay

To characterize yeast transition inside the *A. castellanii* trophozoites, we utilized a method that we previously validated (Pruksaphon et al., 2020), with modifications. Briefly, *A. castellanii* cells were counted and cell viability was determined as described above. Thereafter, the cells were suspended and adjusted to 10^6^ cells/ml in 1 ml PBS and added to 6-well tissue culture plate (Corning). The plates were incubated at 37°C for 2 h to allow for *A. castellanii* acclimation. *T. marneffei* conidia were added to *A. castellanii* at a MOI of 10 for 2 h. After removing non-internalized conidia with PBS, *A. castellanii* cells were cultured with fresh PBS for 48 h at 37°C. Next, the infected *A. castellanii* cells were fixed for 15 min with 4% formaldehyde in cold PBS. The *A. castellanii* cells were suspended in 1%Triton-X 100 in deionized water and permeabilized by shear stress induced by pulling the suspension through a 27-gauge needle (Moffat and Tompkins, 1992). Suspended cells were washed with cold PBS five times and incubated 2 h at 37°C with 0.5 mg/ml of monoclonal antibody 4D1 (MAb 4D1), a yeast phase specific monoclonal antibody of *T. marneffei* mannoprotein (Pruksaphon et al., 2020). The cell suspension was washed five times with 2% BSA-PBS and then incubated for 2 h with Alexaflor 555 labeled goat anti-mouse IgG (Molecular Probes, Eugene, OR, USA) in a 1:500 dilution. Suspended cells were then washed five times with cold PBS. Thereafter, fungal cells were also labeled with Calcofluor white (Sigma, St. Louis, MO, USA) (diluted 1:20 in PBS) for 5 min before a final wash in cold PBS. The samples were imaged using a Nikon DS Fi1 (Nikon, Tokyo, Japan).

### Determination of *T. marneffei* melanin production within *A. castellanii* using an indirect immunofluorescence assay

The method for preparing *T. marneffei* infected *A. castellanii*, MOI, fixation and permeabilization were carried out as detailed above. After permeabilization, the cell suspensions were incubated with 10 mg/ml of MAb 8D6 (anti–fungal melanin monoclonal antibody) for 2 h at 37°C (Youngchim et al., 2004; Chongkae et al., 2021). Then, the samples were washed five times with 2% BSA-PBS and then incubated for 2 h with Alexa Fluor 488 conjugated goat anti-mouse IgM antibody (Molecular Probes, Eugene, OR, USA) in a 1:500 dilution. Suspended cells were then washed five times with cold PBS. Next, fungal cells were labeled with Calcofluor white as detailed above and washed. A negative control used only conjugated goat anti-mouse IgM without the primary melanin-binding MAb. The images were acquired using a Nikon DS Fi1.

### Co-culture experiment with *T. marneffei* and cellular supernatant of *A. castellanii*

*A. castellanii* was harvested from tissue culture flasks, washed with PBS, and centrifuged at 3,000 rpm for 10 min. Then, 1×10^7^ cells/ml of *A. castellanii* were mechanically broken with 0.5-mm glass Ballotini beads (BioSpec, Inc.) in a homogenizer (BioSpec, Bartlesville, OK, USA) with a protease inhibitor cocktail (Sigma). The homogenate was then centrifuged at 10,000 rpm for 30 min at 4°C and the cellular supernatant was decanted from the mixture solution. Protein concentration was measured by the Coomassie Brilliant Blue G-250 binding method (Bio-Rad Labs, Hercules, CA, USA) (Bradford, 1976). For co-culture study, *T. marneffei* conidia (1 ml of 1×10^7^ conidia/ml) were inoculated into 1 ml of cellular supernatant of *A. castellanii* in six-well tissue culture plates. After 48 h incubation at 37°C, *T. marneffei* cells were stained with anti-melanin MAb 8D6. In addition, the phase transition in *T. marneffei* was not only observed in fission yeast but also confirmed by staining with the yeast phase-specific MAb 4D1 as previously described (Pruksaphon et al., 2020). *T. marneffei* control wells (PBS without cellular supernatant of *A. castellanii*) were included in this study.

### Statistical analysis

Student’s *t* test was used for statistical analyses. Both the statistical analysis and the graphs were compiled by two tailed, unpaired Student’s t-test using Prism 5 software (GraphPad). A *P*-value < 0.05 was considered significant.

## Results

### Phagocytosis of *T. marneffei* and *C. albicans* by *A. castellanii*

**Figure 1:**
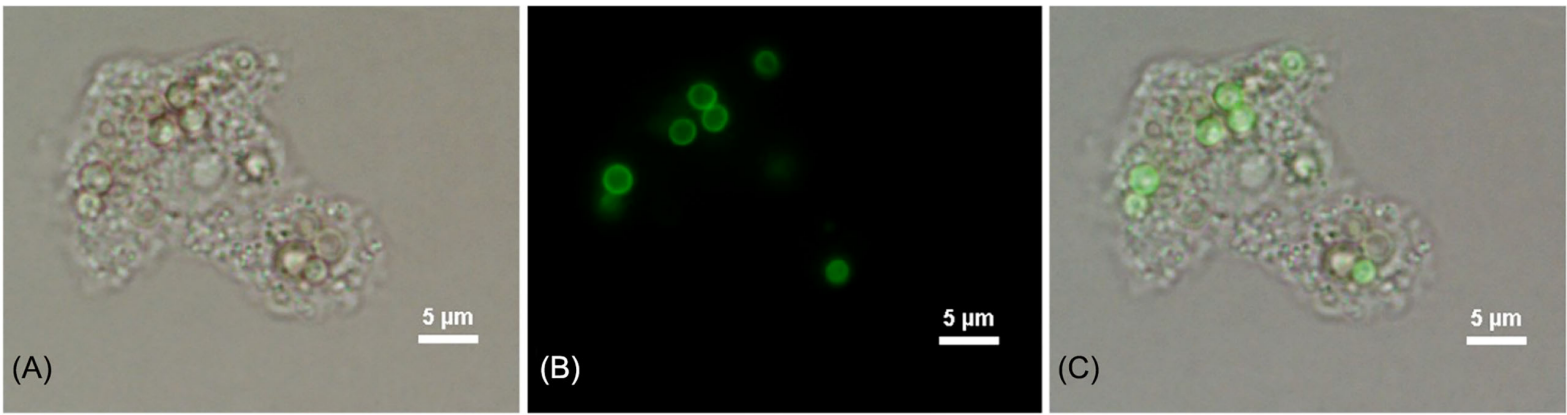
Phagocytosis image of 2 h post-incubation of *A. castellanii* with FITC-labeled *T. marneffei* conidia. Corresponding bright fields **(A)**, fluorescence panel **(B)** and a merged channel showing the overlapping of images **(C)**. The picture was taken under 1,000x magnifications with a Nikon Eclipse 50i fluorescence microscope.

**Figure 2:**
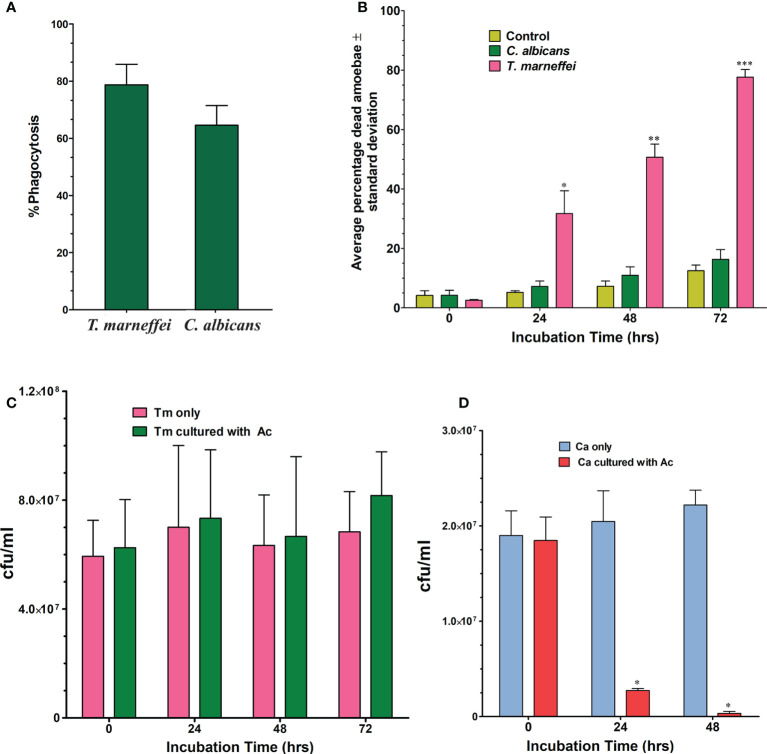
Phagocytosis of *T. marneffei* (Tm) and *C. albicans* (Ca) by the *A. castellanii* (Ac). **(A)** The percentage of phagocytosis was determined by counting the total number of *A*. *castellanii* with internalized conidia or yeast cells per 100 **(A)** *castellanii* cells after 2 h of co-incubation. The bars represent the mean and SD from three independent experiments. **(B)** Average percentage of dead amoebae (*A. castellanii*) after co-incubation with *C. albicans* or *T*. *marneffei* at different time points, 0, 24, 48 and 72 h Amoebae incubated in PBS alone was included in the experiment as negative control. When *T. marneffei* was compared to *C. albicans*, there was a significant difference at 24, 48, and 72 hours. *p = 0.0002 at 24 h, **p = 0.0002 at 48 h and ***p = 0.0001 at 72 h **(C)** *T. marneffei* viability in the absence or in the presence of amoebae at 37°C. There was no significant statistical difference. **(D)** *C. albicans* viability in the absence or in the presence of amoebae at 37°C. There are significant differences in *C*. *albicans* co-incubated with amoebae compared to the control (in PBS). (*p < 0.0001).

The percentage of phagocytosis for *A. castellanii* in co-culture with *T. marneffei* conidia was determined using bright field and fluorescence microscopy (**Figure 1**). After incubation for 2 h at 37°C, the percentage of phagocytosis of *A. castellanii* with *T. marneffei* conidia was 78.8% and the efficiency of this phagocytosis was higher than that observed in yeast cells of *C. albicans*, 64.6%, although this difference was not statistically significant (**Figure 2A**).

### Amoebae were killed by *T. marneffei* and *C. albicans*

Trypan blue exclusion assays were used to determine the percentage of amoebae alive after incubation with the fungi. The results, shown in **Figure 2B**, revealed that 31.7% (*p*=0.005) of amoebae exposed to *T. marneffei* were dead at 24 h of co-culture when compared to the control (*A. castellanii* only, 5.2%), or co-culture with *C. albicans* (7.20%). At 48 h, there were significantly more dead amoebae found from the co-culture with *T. marneffei*, which increased to 50.7% (*p*= 0.0002) compared to 7.3% with the control or 10.9% in the co-culture with *C. albicans*. At 72 h, the number of dead amoebae in co-culture with *T. marneffei* increased to 77.7% (*p*=0.0001) compared to 12.5% and 16.3% of dead amoebae in the control and in the presence of *C. albicans*, respectively.

### Co-culture experiment of *T. marneffei* and *C. albicans* with amoebae

Incubation of *T. marneffei* with amoeba cells at different times was determined by counting CFUs. Although the number of CFUs of *T. marneffei* was higher than in the control (without *A. castellanii*), the difference was not statistically significant (**Figure 2C**). In contrast, the viability of *C. albicans* after co-culture with *A. castellanii* was significantly decreased at 24 and 48 h of incubation, *p*<0.0001 (**Figure 2D**).

### Electron microscopy of *T. marneffei* –amoeba interaction

**Figure 3:**
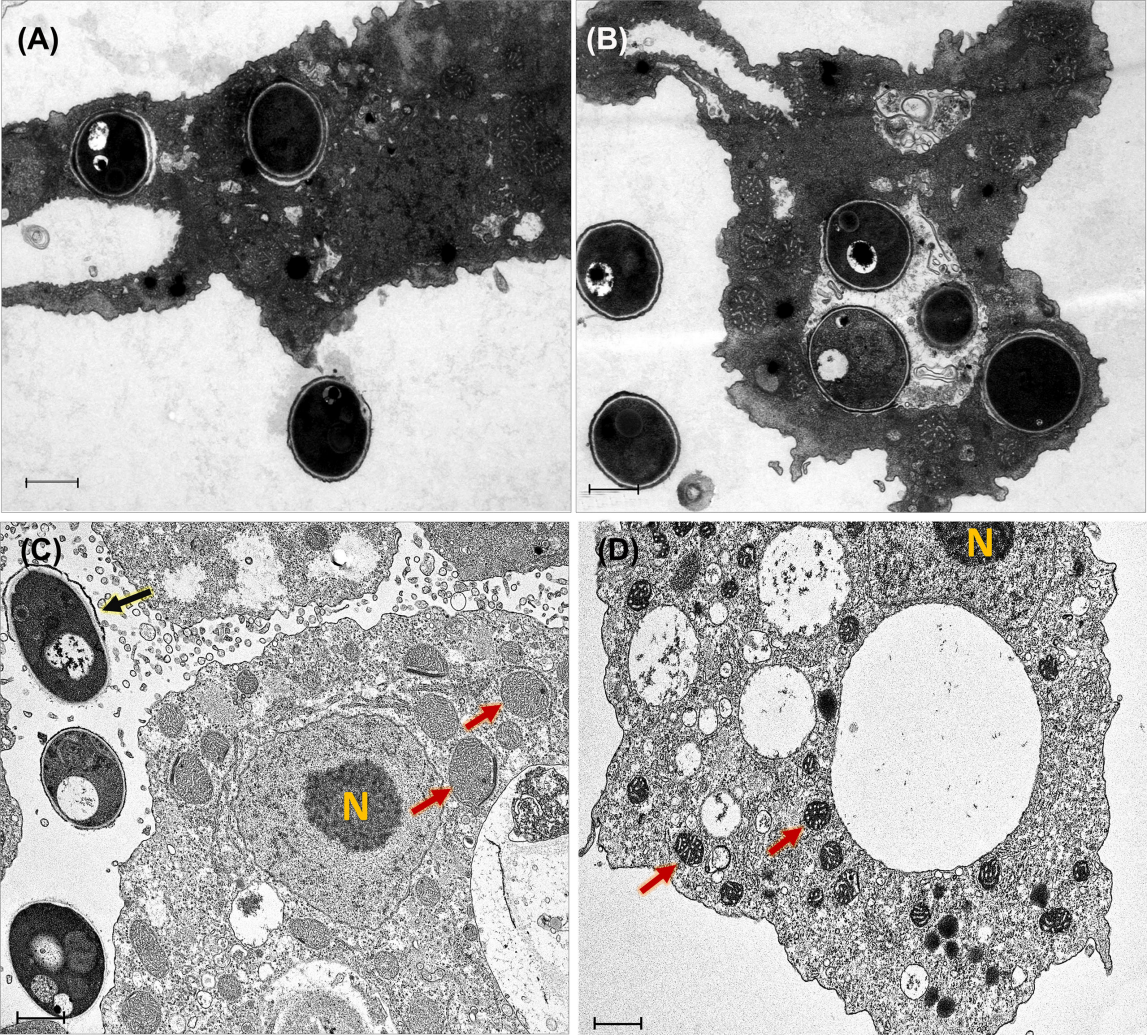
Transmission electron micrographs of *T. marneffei* interacting with *A*. *castellanii* trophozoites after co-cultivation at 37°C. The conidia of *T. marneffei* are phagocytosed and enclosed in a membrane bound vacuole of amoeba cell after 30 minutes **(A)** and 2 h **(B)** post-infection. *T. marneffei* yeast cell (depict by black arrow) interacted with amoeba manifest reduced cytoplasmic electron density and caused swelling of *A*. *castellanii* mitochondria (depicted by two red arrows) at 48 h post-infection, these phenomena indicated the amoeba cell injury **(C)**. The control *A*. *castellanii* trophozoites without *T. marneffei* infection, the two red arrows represented the normal appearance of mitochondria **(D)**. The photograph was taken under 3,000x magnifications with a JEM-2200FS electron microscope. N represents the *A*. *castellanii* nucleus and the scale bars represented 1 μm.

The pictures obtained by the transmission electron microscope provided more detailed information of the cytopathic effect on *A*. *castellanii* trophozoites induced by *T. marneffei* infection. TEM ultrastructure was used to demonstrate that amoebae internalized *T. marneffei* conidia. At the early phase of internalization (30 min and 2 h) the fungal conidia were phagocytosed and enclosed in membrane-bound vesicles inside *A. castellanii* with the typical morphology present of amoeba. The amoebae had more than one internalized fungal cell and individual fungal conidia in separate phagocytic vesicles indicating two independent phagocytic circumstances (**Figures 3A, B**). At 48 h infection, a swelling of amoeba cells was observed, which appear also devoid of pseudopodia indicating the impairment of osmotic balance (Ferro et al., 2009). Moreover, the interaction of *T. marneffei* with the amoeba results in swelling of mitochondria and reduced the amoeba cytoplasmic electron density corresponding with amoeba cell injury (Steenbergen et al., 2004), The extracellular yeast cell of *T. marneffei* was presented suggesting the *A. castellanii* cells lysis (**Figures 3C, D**).

### Yeast phase transition and melanin synthesis in *T. marneffei* during co-culture with amoebae

**Figure 4:**
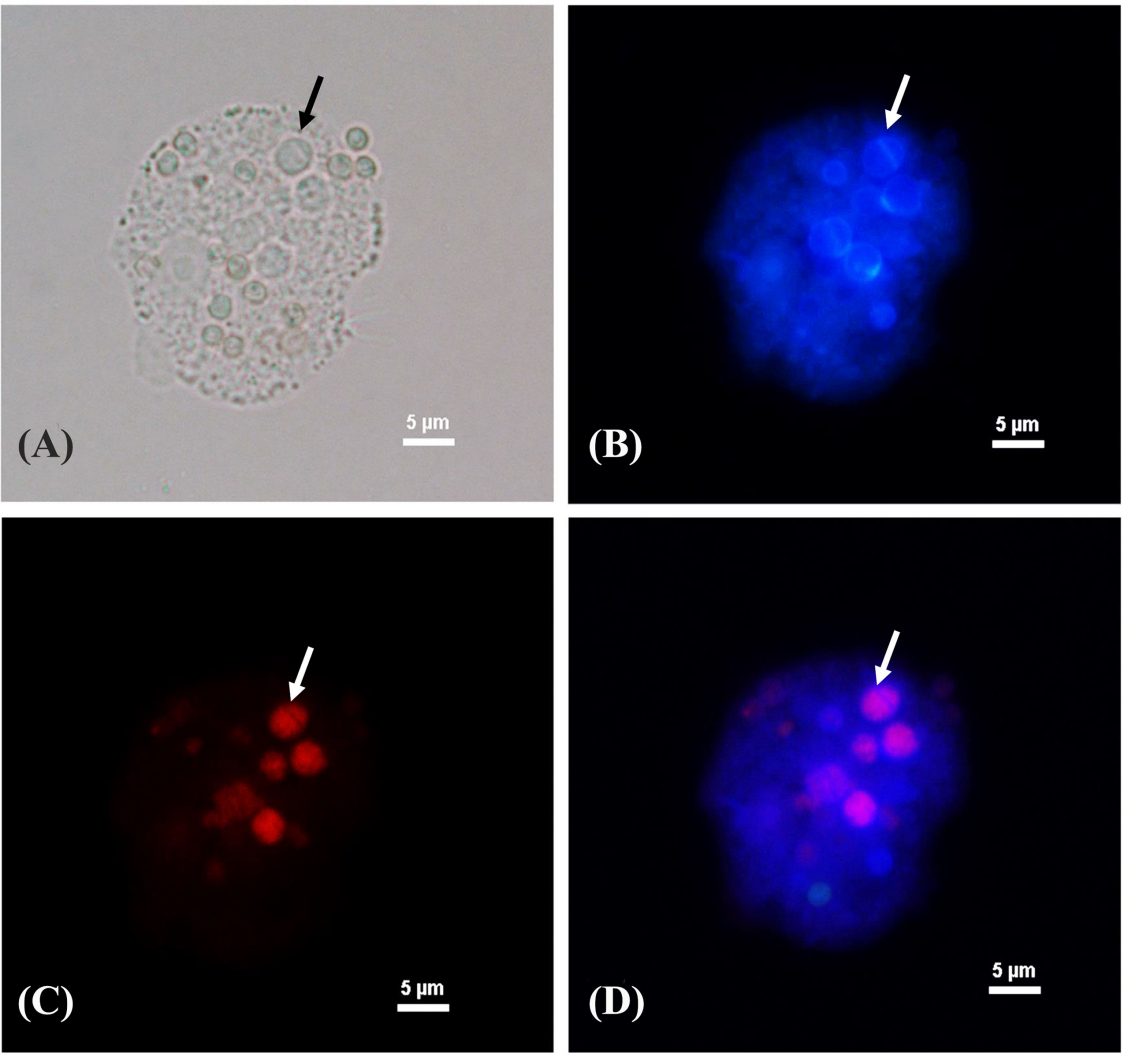
Phase transition of *T. marneffei* interacting with *A*. *castellanii* after co-cultivation at 37°C for 48 h The corresponding bright fields **(A)**, Calcofluor white labeled yeast cells **(B)**, The red signal of *T. marneffei* yeast cells immunostaining with yeast phase-specific mannoprotein antibody (MAb 4D1) and Alexa fluor 555 conjugated goat anti-mouse IgG **(C)**, and the merged signals of the panel B and panel C show the co-localized signal as purple **(D)**. The arrow indicates the fission yeast cell of *T. marneffei*. The picture was taken under 1,000x magnifications with a Nikon Eclipse 50i fluorescence microscope.

**Figure 5:**
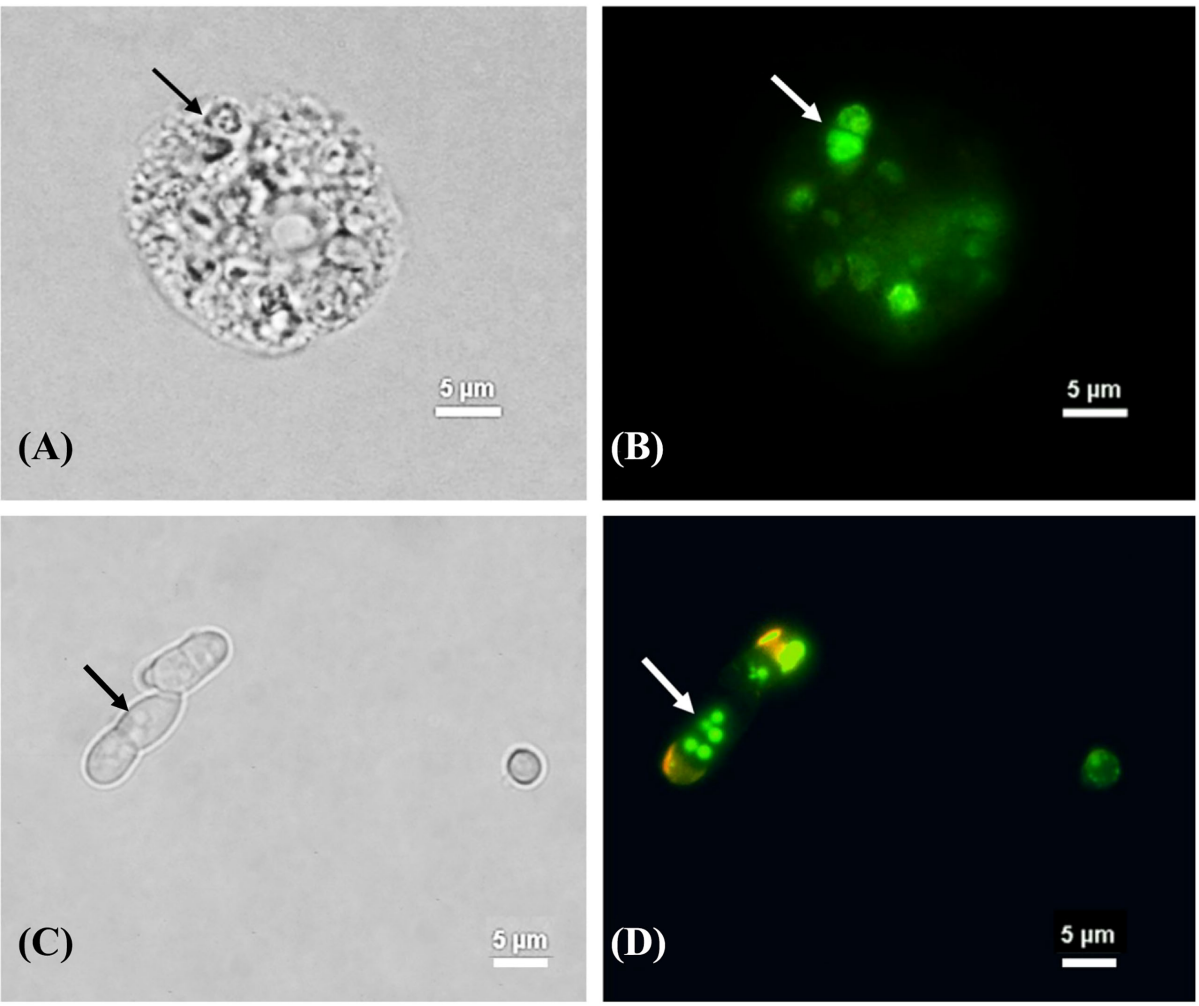
The melanization of *T. marneffei* interacting with *A*. *castellanii* after co-cultivation at 37°C. The corresponding bright field **(A, C)** and immunofluorescence **(B, D)**, the images showing the green signal of *T. marneffei* yeast cells immunostaining with anti- fungal melanin antibody (MAb 8D6) and Alexa fluor 488 conjugated goat anti-mouse IgM after 48 h co-incubation with *A*. *castellanii*. The arrows indicated fission yeast cells **(A, B)** and the melanin granules of *T. Marneffei* **(C, D)**. The picture was taken under 1,000x magnifications with a Nikon Eclipse 50i fluorescence microscope.

After *T. marneffei* interacted with *A. castellanii* at 37°C for 48 h, *T. marneffei* was transformed to yeast phase showing fission yeast and confirmed by demonstrating positive labeling with MAb 4D1, a yeast phase specific MAb of *T. marneffei* mannoprotein (**Figure 4**). *T. marneffei* did not only undergo morphogenesis to a fission yeast, the yeast forms also produced melanin as they were stained with the anti-melanin MAb 8D6 (**Figure 5**). Melanin granules were clearly detectable in yeast cell of *T. marneffei* (**Figures 5C, D**). In addition, yeast transition and melanin synthesis were seen in *T. marneffei* during co-culture with cellular supernatant of *A. castellanii* to confirm that *T. marneffei* can utilize the nutritional sources inside the amoeba cells for the development of virulence attributes (**Supplementary Figure 1**). *T. marneffei* conidia were unable to transform into fission yeast after incubation in PBS at 37°C and were stained negative with MAb 4D1.

## Discussion

Amoebae represent an important class of environmental predators in soils and may impose selection pressures on environmental populations of microbes for them to develop variations with mammalian pathogenicity (Mylonakis et al., 2002; Steenbergen et al., 2003). The interaction of the dimorphic fungus *T. marneffei* with the free-living amoeba *A. castellanii* is documented for the first time in our findings, demonstrating that *A. castellanii* can serve as an alternative host system for this fungus.

Our results found that *A. castellanii* effectively ingests *T. marneffei* conidia, which resulted in amoebal death 31.7%, 50.7% and 77.7% at 24, 48 and 72 h, respectively (**Figure 2B**). The results demonstrated a significant decrease in viable amoebae indicating that *T. marneffei* was capable of killing amoebae and exploiting them for sustenance (**Supplementary Figure 1**). *Caenorhabditis elegans* was previously used to investigate the virulence of *T. marneffei*, and it was discovered that a *T. marneffei* strain generating red pigment could kill the nematodes faster than the fungal strain that cannot produce red pigment (Huang et al., 2014). This suggested that, in this context, the red pigment may be involved in *T. marneffei* pathogenicity. In contrast, our study showed that *C. albicans* was rapidly killed by *A. castellanii* (**Figure 2C**), which is similar to that seen with *Saccharomyces cerevisiae* as *S. cerevisiae* reduced more than half of the yeast cells after 90 min of co-incubation with amoebae. Our finding that *A. castellanii* can feed on *C. albicans* is consistent with findings previously reported in *C. neoformans* (Castellani, 1955) and subsequently demonstrated to feed on *Torulopsis famata* and *Candida parapsilosis* (Nero et al., 1964).

Adhesion is an important factor that occurs prior to phagocytosis. Previous studies have revealed that the mannose binding protein (MBPs) on *A. castellanii* cell surface plays an important role in fungal recognition by amoeba (Goncalves et al., 2019). Moreover, mass spectrometry and in silico alignment revealed highly conserved portions between mouse macrophage (RAW 264.7) receptors and amoeboid receptors, suggesting that pathogen detection systems may have evolved in concert in both phagocytic cells (Ferreira et al., 2022). Our electron microscopy results reveal that *T. marneffei* are enclosed in membrane-bound vacuole after ingestion by amoebae (**Figure 3**). For this fungus, our data demonstrates that internalized fungal cells can exploit the amoebae after ingestion as well as gain nutrients by feeding from the remains of the killed host cells. Our data also suggests that the mechanism of *T. marneffei* killing of amoeba cells required contact between the fungal and amoebal cells.

When other thermally dimorphic fungi, such as *B. dermatitidis*, *H. capsulatum*, and *Sporothrix* spp., were co-incubated with *A. castellanii* at 37°C, some yeast cells of these dimorphic fungi switched to filamentous forms, which is usually a temperature permissive for yeast growth (Steenbergen et al., 2004). In contrast, our findings demonstrated a shift from the conidial to fission yeast form of *T. marneffei* after interaction with amoebae at 37°C for 48 h, as confirmed by yeast phase specific MAb 4D1 (**Figure 4**). Culturing *T. marneffei* conidia with amoebae extracts at 37°C also led to morphogenic change to the fission yeast form. Indeed, the morphological transition of dimorphic fungi from mold to yeast is crucial to promote pathogenicity *via* phagocytosis escape, regulation of the cytotoxic environment of the phagolysosome, and increased reactive oxygen species (ROS) degradation (Klein and Tebbets, 2007; Boyce and Andrianopoulos, 2015; Pruksaphon et al., 2020; Hoft et al., 2022).

We also provided evidence that *T. marneffei* can produce melanin after interacting with *A. castellanii* suggesting that *T. marneffei* can exploit amoeboid nutrition as melanin substrate (**Figure 5** and **Supplementary Figure S1**). In fact, *T. marneffei* can produce DOPA (dihydroxyphenylalanine) melanin in yeast cells, which require either L-DOPA or tyrosine as an external substrate (**Supplementary Figure S2**) (Liu et al., 2014). *A. castellanii* contains certain precursors for the various melanin production pathways including tyrosine residues (Clarke et al., 2013). As a result, it is likely that tyrosine provides as a substrate for melanin production in *T. marneffei*. Tyrosine can be hydroxylated by tyrosinases and/or laccases to generate DOPA melanin (Langfelder et al., 2003). The expression of genes in the *T. marneffei* tyrosine catabolic gene cluster indicates the numerous functions that these genes play not only in adapting to the nutritional sources available but also in the synthesis of protective melanin (Boyce and Andrianopoulos, 2015). However, further study is required to determine whether tyrosine serves as precursor of melanization of *T. marneffei* during interaction with amoebae.

Fungal melanin serves a crucial role in protecting fungi against harsh environmental conditions such as high temperatures and desiccation, radiation, and oxidative and osmotic stress (Cordero and Casadevall, 2017; Liu et al., 2021). Furthermore, melanin is linked to the virulence and pathogenicity of various human pathogenic fungus, most notably in *A. fumigatus* and *C. neoformans* (Smith and Casadevall, 2019). According to our findings, melanization in *T. marneffei* yeast cells was detected not only within the cell wall, but also in the cytoplasm as melanin granules after co-cultured with amoebae (**Figure 5**). TEM has previously demonstrated the presence of melanin granules in the innermost layer of the cell wall of *T. marneffei* yeast cells in the form of a beaded arrangement (Liu et al., 2014).

Recent studies with melanized conidia of *A. fumigatus* showed lower internalization rates by *Dictyostelium discoideum*, a soil-dwelling social amoeba than melanin-deficient conidia. Indeed, melanin in *A. fumigatus* conidia appeared to suppress acidification of phagolysosomes in amoebas and macrophages, allowing the melanized fungus to survive longer within these phagocytes than the melanin-deficient mutant (Thywissen et al., 2011; Ferling et al., 2020). Furthermore, melanin is an important determinant of fungal interaction with the innate immune system, and it can enable *A. fumigatus* escape and survive in macrophages by impeding phagosome formation (Akoumianaki et al., 2016) and acidification of phagolysosomes (Ferling et al., 2020), as well as preventing phagocyte apoptosis (Volling et al., 2011). These results support the postulate that melanization of *T. marneffei* yeast enhances its pathogenesis in environmental predators like *A. castellanii*, which is consistent with data on melanized *T. marneffei* and mouse macrophages (Liu et al., 2014).

Since dimorphic switching and melanin producing are major virulence factors in *T. marneffei*, this is significant evidence that the development and conservation of virulence traits in *T. marneffei* are not always linked to mammalian (e.g. bamboo rat and human) contact (Pruksaphon et al., 2022), but rather can also evolve as a result of selection pressures caused by interactions with environmental predators in the soil. Similarly, many characteristics of the interactions between fungal pathogens and amoebae are related to those of other phagocytic mammalian cells; these fungal virulence factors appear to play a role in pathogenicity to various environmental hosts, as well as during mammalian infections (Casadevall et al., 2019; Ferreira et al., 2022).

In conclusion, our findings show that *A. castellanii* can phagocytose *T. marneffei* conidia in a similar manner to that occurring with macrophages and that virulence features such as dimorphic switching and melanin, which are required during mammalian infections, appear to be important for protection against amoeba killing. In addition, investigating how this and other fungi interact with soil amoebae at the molecular level is a promising strategy for deciphering evolutionary connections and the origins of virulence in fungi.

## Data availability statement

The original contributions presented in the study are included in the article/**Supplementary Material**. Further inquiries can be directed to the corresponding author.

## Author contributions

KP, JN, and SY contributed to the investigation, the methodology, and the writing of the original draft. KP, JN, PT, MP, and SY contributed to the conceptualization, the methodology, the scientific advice, the project administration, and editing of the manuscript. All authors contributed to the article and approved the submitted version.

## Funding

This study was supported by a research grant from Chiang Mai University, Chiang Mai, Thailand (Post- doctoral Fellowship, Fiscal Year 2022) and the Thailand Research fund (TRF) grant number MRG 4980060.

## Conflict of interest

The authors declare that the research was conducted in the absence of any commercial or financial relationships that could be construed as a potential conflict of interest.

## Publisher’s note

All claims expressed in this article are solely those of the authors and do not necessarily represent those of their affiliated organizations, or those of the publisher, the editors and the reviewers. Any product that may be evaluated in this article, or claim that may be made by its manufacturer, is not guaranteed or endorsed by the publisher.
