# Supplementary material for: Interaction of Talaromyces marneffei with free living soil amoeba as a model of fungal pathogenesis
Source: Front Cell Infect Microbiol. 2022 Oct 3;12:1023067. doi: 10.3389/fcimb.2022.1023067 (PMC9574045; doi:10.3389/fcimb.2022.1023067)
Supplement: Supplementary file 1 [file DataSheet_1.docx]

Supplementary Material

## Supplementary Figure


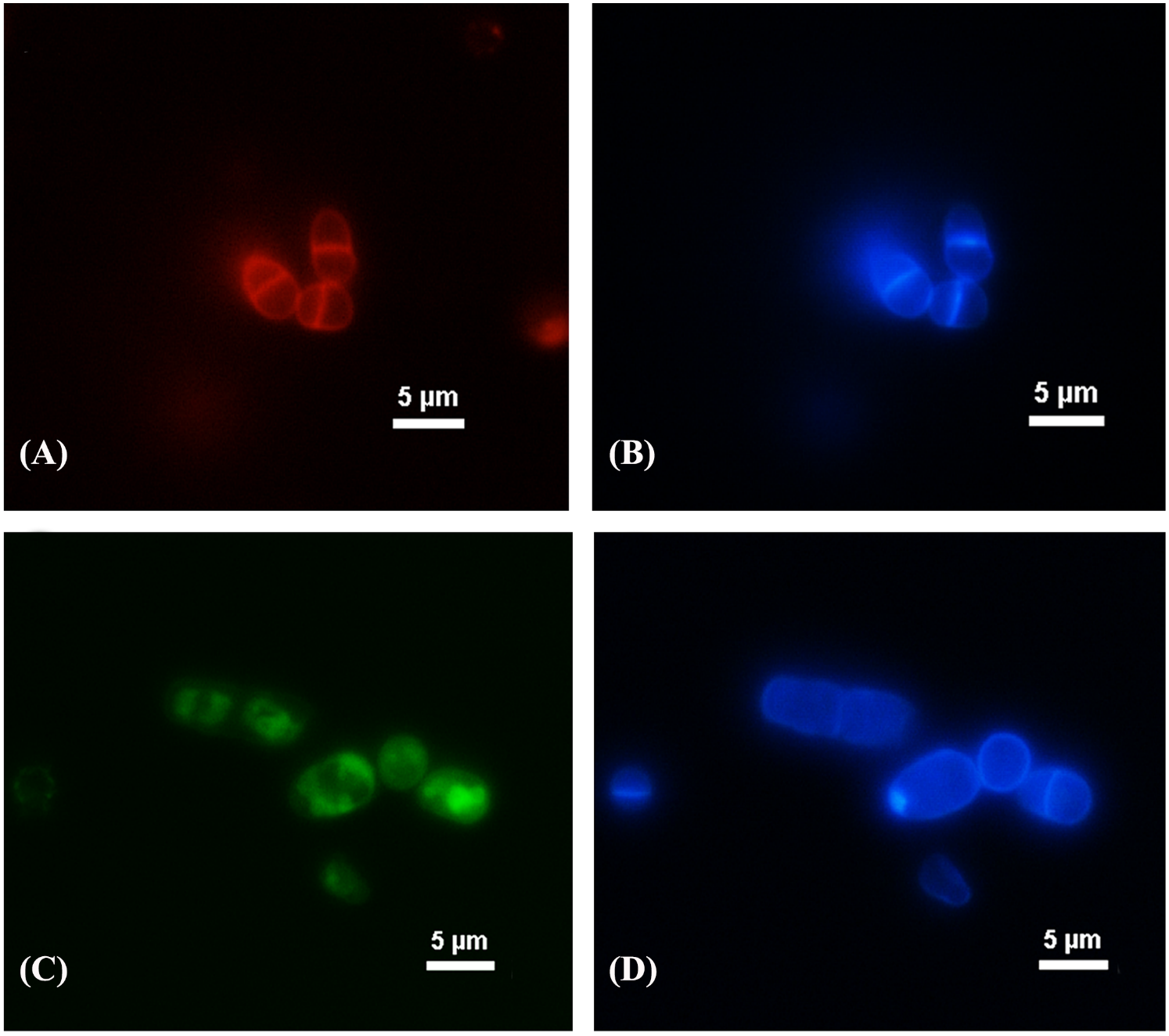


**Supplementary Figure 1**. Induction of *T. marneffei* phase transition from conidia to yeast with the cellular supernatant of *A. castellanii* at 37 ^o^C for 48 h. The corresponding immunofluorescence (A, C) and Calcofluor white staining (B, D) microscopy images. In (A) and (C), the immunofluorescent labeling was performed using yeast-specific monoclonal antibody (MAb 4D1 pairing with Alexa fluor 555 conjugated goat anti-mouse IgG) and anti- fungal melanin (MAb 8D6 pairing with Alexa fluor 488 conjugated goat anti-mouse IgM), respectively. (Magnifications: 1,000x)


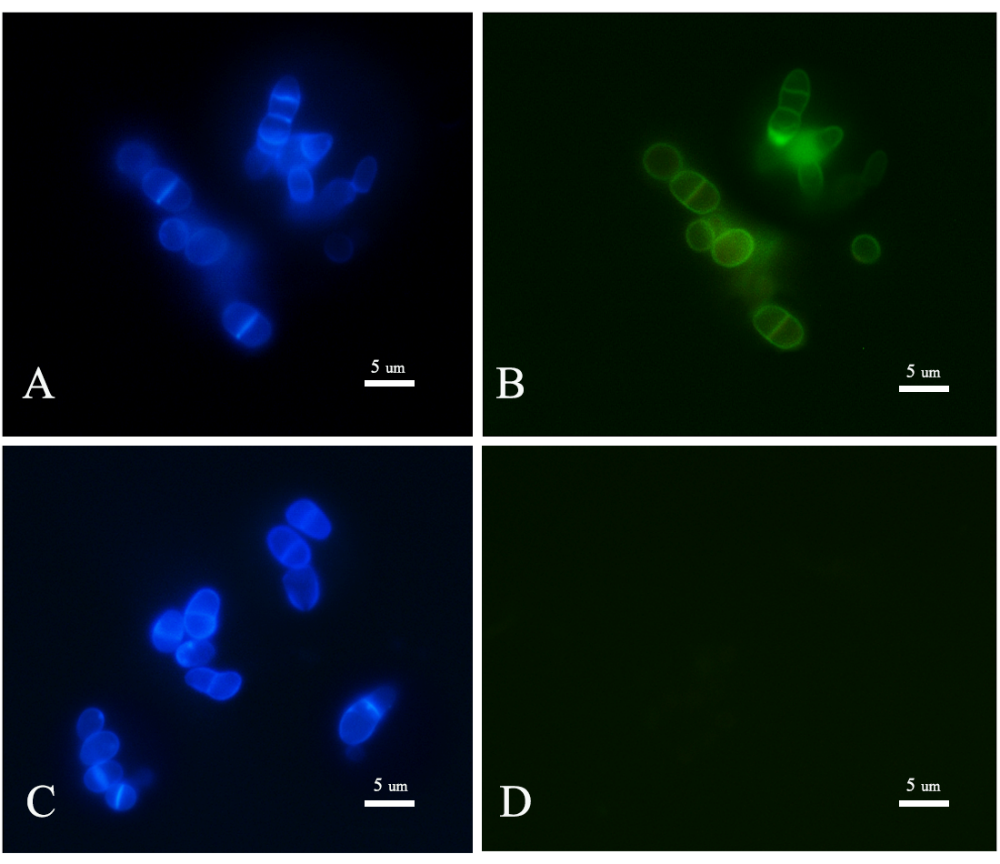


**Supplementary Figure 2.** *T. marneffei* melanization in minimal medium (MM) with or without L-DOPA at 37^o^C. The corresponding Calcofluor white staining (A, C) and the immunofluorescent staining by using MAb 8D6 pairing with Alexa fluor 488 conjugated goat anti-mouse IgM (B, D), respectively. Yeast cells of T. marneffei culturing with L-DOPA were reactive against MAb 8D6 (A, B). In contrast, culturing *T. marneffei* in MM without L-DOPA was not reactive to MAb 8D6 (C, D). (Magnifications: 1,000x)
